# Sports participation and obesity incidence in chinese adolescents: a 7-year prospective cohort study

**DOI:** 10.1038/s41598-026-50212-y

**Published:** 2026-05-11

**Authors:** Derun Qiu, Chunmin Dai

**Affiliations:** 1 College of P.E and Sports, Beijing Normal University, Beijing, Beijing, 100875 China; 2 International Football Education School, Jilin Agriculture University, Changchun, 130118 Jilin China

**Keywords:** Adolescent obesity, Sports participation, Prospective cohort study, Dose-response relationship, Physical activity, Cox proportional hazards model, Diseases, Health care, Medical research, Risk factors

## Abstract

Adolescent obesity has risen to become a critical public health challenge around the world. According to the World Health Organization, roughly 340 million children and adolescents aged 5–19 were overweight or obese by 2016, representing a nearly sixfold increase since 1975. In China, rapid urbanization and shifting dietary patterns have accelerated this trend among school-age populations. Although physical activity is broadly considered protective against weight gain, longitudinal evidence that quantifies the magnitude of sports participation’s effect on obesity incidence during adolescence remains sparse. We aimed to examine the dose-response relationship between sports participation intensity and seven-year obesity incidence among Chinese adolescents, and to identify subgroup-specific effect modification by sex, age, and baseline body mass index. We conducted a prospective cohort study drawing on data from the National Adolescent Health Surveillance System. A total of 89,532 initially non-obese Chinese adolescents aged 12–15 years were followed from 2016 to 2023. Sports participation intensity was assessed biannually through validated questionnaires, and obesity incidence was ascertained using WHO age- and sex-specific BMI criteria. Cox proportional hazards regression models were fitted with adjustment for demographic, socioeconomic, and time-varying behavioral confounders. Propensity score matching and marginal structural models served as supplementary analytical approaches to strengthen causal inference. Over 523,847 person-years of observation, 8,947 incident obesity cases were identified (17.1 per 1,000 person-years). Compared with minimal physical activity, high sports participation (≥ 300 min/week) was associated with a 58% lower obesity risk (HR = 0.42, 95% CI 0.37–0.48), and a clear dose-response gradient was observed. Moderate and low participation showed intermediate protective effects (HR = 0.61 and 0.73, respectively). These associations held up across multiple sensitivity analyses and were stronger among boys than girls. Sustained sports participation during adolescence substantially reduces obesity incidence in a dose-dependent manner. These findings offer compelling support for physical activity promotion policies and school-based interventions targeting this critical developmental period. This study was reported following the STROBE guidelines for observational studies.

## Introduction

The escalating prevalence of childhood and adolescent obesity stands as one of the most pressing public health concerns of the 21st century, reshaping pediatric medicine and preventive health strategies across the globe^1^. Epidemiological data paint a stark picture: approximately 340 million children and adolescents aged 5–19 were classified as overweight or obese worldwide by 2016, a nearly sixfold increase from 1975^2^. In China specifically, the prevalence of overweight and obesity among children and adolescents has surged from under 5% in the 1990s to over 20% in recent national surveys, driven by rapid urbanization, dietary shifts toward energy-dense processed foods, and declining habitual physical activity^3,4^. This upward trajectory carries consequences that extend well beyond childhood, as adolescent obesity tracks strongly into adulthood and is associated with elevated risks of type 2 diabetes, cardiovascular disease, certain cancers, and reduced life expectancy^5^.

Physical activity participation is widely recognized as a cornerstone intervention for obesity prevention and management in young populations, yet the precise magnitude of its protective effect remains a matter of ongoing scientific inquiry^6^. Cross-sectional studies have yielded inconsistent findings, largely owing to limited sample sizes, heterogeneous measurement methods, and an inability to establish temporal precedence. Several cohort investigations conducted in diverse geographical settings have attempted to quantify the dose-response relationship between physical activity levels and adiposity markers, but methodological heterogeneity and short follow-up periods have precluded definitive conclusions^7^. Furthermore, much of the existing evidence relies on self-reported physical activity assessments, which introduce recall bias and measurement error that may obscure genuine associations^8^.

Beyond measurement challenges, few studies have adequately accounted for the complex confounding structure inherent in the activity–obesity relationship, including socioeconomic conditions, dietary habits, genetic susceptibility, and the built environment. The temporal dimension presents an additional hurdle: most published evidence derives from snapshot observations rather than extended longitudinal follow-up capable of capturing the behavioral shifts and developmental transitions characteristic of adolescence. These gaps call for research designs that combine large sample sizes with prolonged observation and rigorous statistical adjustment.

The present investigation was designed to address these limitations through a prospective cohort study following nearly 90,000 Chinese adolescents over seven years. We employed Cox proportional hazards models with time-varying exposure and covariate adjustment, supplemented by propensity score matching and marginal structural models, to estimate the association between sports participation intensity and obesity incidence^9^. Our study makes several contributions to the literature. First, the use of objectively measured anthropometric data integrated with comprehensive physical activity records reduces the information bias that plagues many prior investigations. Second, the extended follow-up enables examination of delayed effects and identification of critical periods of susceptibility. Third, subgroup analyses and sensitivity testing strengthen causal inference and illuminate population-level heterogeneity^10^. Taken together, these features position our work to generate actionable evidence for targeted intervention strategies and public health programming aimed at curbing the adolescent obesity epidemic.

## Theoretical framework and literature review

### Measurement theory of adolescent sports participation

Sports participation, defined as the volitional engagement in structured or unstructured physical activities that elevate energy expenditure above resting metabolic levels, is a multidimensional construct^11^. Its measurement traditionally encompasses four cardinal dimensions: frequency (the number of discrete activity episodes within a given time window), intensity (the physiological demand, commonly expressed in metabolic equivalent values, METs), duration (the temporal length of each exercise bout), and activity type (distinguishing aerobic, resistance, flexibility, and sport-specific movements)^12^. The total physical activity volume integrates these dimensions. The relationship can be expressed as PAvolume = Σ(F_i_ × I_i_ × D_i_), where F, I, and D represent frequency, intensity, and duration for activity type i, respectively.

Standardized instruments such as the International Physical Activity Questionnaire (IPAQ) and the Physical Activity Questionnaire for Adolescents (PAQ-A) operationalize these dimensions into comparable metrics across populations^13^. Recent modifications have introduced weighted scoring systems that account for differential health benefits across activity types, yielding composite scores that weight MET values by activity category. Theoretical frameworks explaining adolescent activity behavior have evolved from simple models toward socio-ecological perspectives. The Youth Physical Activity Promotion Model, for instance, posits that participation arises from the interplay of intrapersonal factors (self-efficacy, enjoyment), interpersonal influences (peer support, parental modeling), and environmental contexts (facility availability, neighborhood safety)^14^.

### Obesity occurrence mechanisms and influencing factors

Obesity is a chronic metabolic disorder characterized by excessive adipose tissue accumulation. The World Health Organization defines childhood and adolescent obesity through age- and sex-specific BMI percentiles, with values exceeding the 95th percentile indicating obesity and those between the 85th and 95th percentiles signifying overweight status^15^. At its core, obesity development follows the principle of energy balance: chronic positive energy balance—where caloric intake exceeds expenditure—drives progressive fat accumulation. Yet this apparently straightforward thermodynamic framework conceals intricate physiological machinery, including the hypothalamic–pituitary–adipose axis that governs appetite regulation through leptin and ghrelin signaling, and peripheral tissue modulation of substrate oxidation and lipogenesis^16^.

The etiology of obesity involves complex gene–environment interactions rather than simple genetic determinism or purely behavioral causation. Genome-wide association studies have identified over 900 loci linked to obesity susceptibility, yet these variants collectively explain only 5–10% of BMI variance, highlighting the substantial role of environmental modulation^17^. Epigenetic modifications established during critical developmental windows mediate how nutritional exposures and physical activity habits translate into metabolic phenotypes. In rapidly urbanizing populations—especially in China—an obesogenic environment characterized by energy-dense diets, sedentary lifestyles, and reduced active transportation interacts synergistically with genetic predisposition to amplify obesity risk^3^. Sex-specific trajectories diverge during puberty owing to hormonal influences on fat distribution and metabolic regulation, and adolescent obesity prevalence shows marked disparities across socioeconomic strata and geographic regions. 

### Cohort study methodology

Prospective cohort designs follow defined populations forward through time to observe how exposures influence outcome incidence, thereby establishing the temporal precedence essential for causal inference^18^. Causal reasoning within this framework relies on counterfactual logic, contrasting observed outcomes against hypothetical scenarios under alternative exposure states. Because only one potential outcome is observed per individual, population-level average treatment effects are estimated instead^19^.

A particular challenge arises from time-dependent confounding, where intermediate variables simultaneously act as confounders and mediators along causal pathways. Traditional regression adjustment fails when confounders at time t are themselves affected by prior exposure. Marginal structural models combined with inverse probability weighting address this limitation by creating pseudo-populations in which exposure appears randomized^19^. In health research, large-scale databases—electronic health records, wearable device data streams, and administrative registries—enable massive sample sizes and granular temporal resolution, though they also introduce complexities around missing data patterns and selection biases that demand sophisticated statistical remedies^20^. 

## Data sources and research methods

### Data sources and sample selection

Our investigation drew upon data from the National Adolescent Health Surveillance System (NAHSS), a comprehensive population-based registry that integrates school health records, annual physical examinations, and sports participation logs across 28 provinces in China^21^. This database architecture links individual identifiers across multiple administrative platforms—including the Student Physical Fitness Monitoring Network and the Regional Healthcare Information System—enabling longitudinal tracking of health trajectories and behavioral patterns. We accessed anonymized records spanning January 2016 through December 2023, encompassing routine anthropometric measurements, validated physical activity questionnaires, and medical diagnoses coded according to International Classification of Diseases standards.

Participant eligibility required enrollment in secondary education institutions (grades 7–12) at baseline, with age between 12 and 15 years, and normal weight status defined as BMI-for-age between the 5th and 85th percentiles according to WHO growth reference standards^22^. We excluded individuals with pre-existing obesity, documented endocrine disorders affecting metabolism (hypothyroidism, Cushing’s syndrome, growth hormone deficiency), physical disabilities limiting exercise capacity, or incomplete baseline anthropometric data. Pregnancies during follow-up and participation in structured weight management interventions were also excluded, given their potential to confound natural obesity development patterns.

**Fig. 1:**
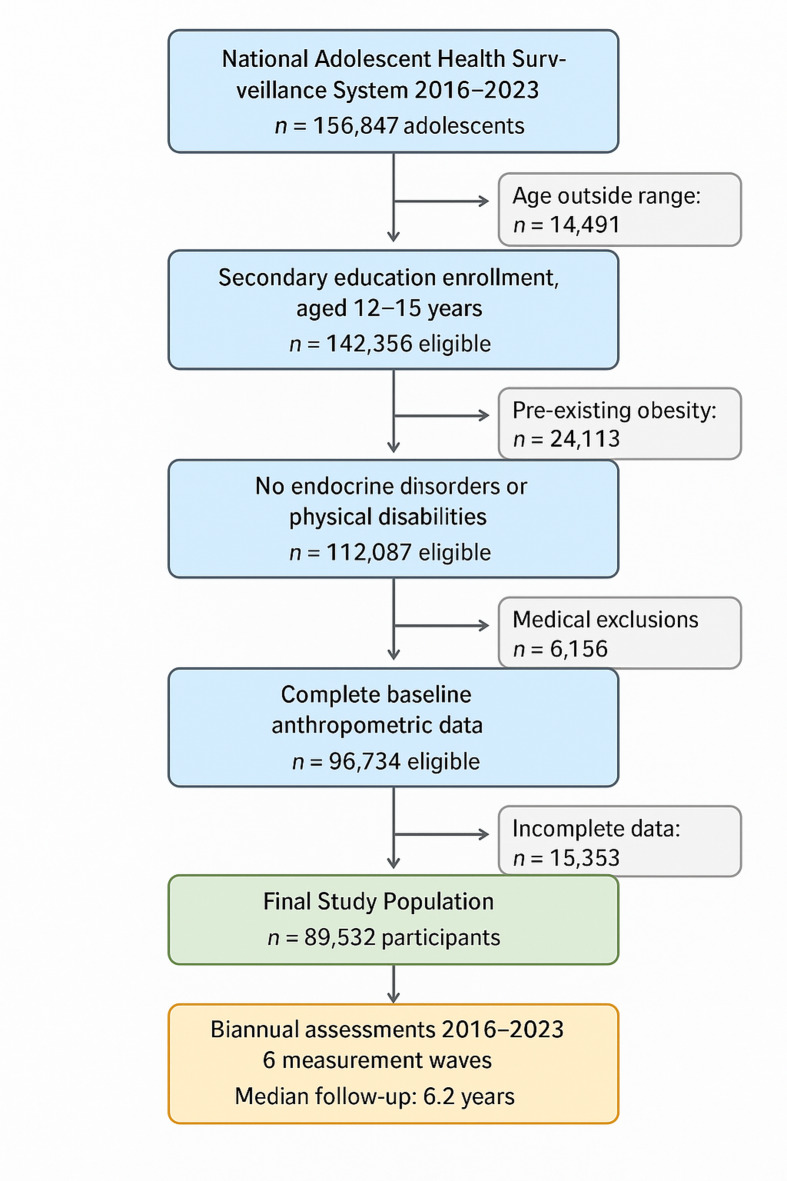
Data collection and participant selection flowchart.

Figure 1 illustrates the systematic screening process that yielded our analytical cohort. From an initial sampling frame of 156,847 adolescents, sequential exclusions removed 67,315 individuals: 28,456 did not meet age criteria, 18,723 had pre-existing overweight or obesity, 6,891 presented documented endocrine or metabolic conditions, 5,432 had physical disabilities restricting exercise capacity, 4,617 lacked complete baseline anthropometric records, and 3,196 were excluded for other reasons (pregnancies, concurrent weight management programs). This process yielded a final analytic sample of 89,532 participants. Baseline data collection took place during September–October of the 2016 academic year, with subsequent biannual assessments through structured school-based health examinations spanning six measurement waves over seven years.

**Table 1:** Baseline characteristics of the study cohort.

Characteristic	Total (*n* = 89,532)	Boys (*n* = 43,586)	Girls (*n* = 45,946)
Age, years (mean ± SD)	13.4 ± 1.1	13.5 ± 1.2	13.3 ± 1.1
Height, cm (mean ± SD)	159.8 ± 9.6	162.3 ± 10.2	157.5 ± 8.4
Weight, kg (mean ± SD)	48.7 ± 8.3	50.2 ± 9.1	47.3 ± 7.2
BMI, kg/m² (mean ± SD)	19.0 ± 2.1	18.9 ± 2.2	19.1 ± 2.0
Urban residence (%)	68.2	67.5	68.9
Parental education ≥ college (%)	54.6	55.1	54.2
Family income > median (%)	51.8	52.3	51.4
High PA (≥ 5 times/week) (%)	42.7	48.9	36.9
Moderate PA (3–4 times/week) (%)	25.8	24.6	26.9
Low PA (< 3 times/week) (%)	31.5	26.5	36.2
Screen time > 2 h/day (%)	47.3	49.6	45.2
Dietary quality score (mean ± SD)	6.8 ± 1.9	6.7 ± 2.0	6.9 ± 1.8
Sleep duration, hours (mean ± SD)	8.2 ± 0.9	8.1 ± 1.0	8.3 ± 0.9
Family history of obesity (%)	23.7	24.2	23.3
Complete follow-up (%)	82.4	81.8	83.0

As presented in Table 1, the cohort showed balanced sex distribution (51.3% female) with a mean age of 13.4 years at enrollment. Participants came predominantly from urban settings (68.2%), reflecting contemporary demographic concentration patterns, though substantial rural representation ensured adequate exposure variability. Quality assurance protocols mandated standardized measurement procedures across all participating sites, with trained personnel conducting anthropometric assessments following unified operational guidelines^23^. Digital calibration of weighing scales and stadiometers occurred quarterly, while data entry underwent automated range checks and logic validations to flag implausible values for manual verification.

### Research design and variable definitions

We adopted a prospective cohort design in which initially non-obese adolescents were tracked across multiple assessment waves to ascertain obesity incidence as a function of baseline and time-varying sports participation patterns. The analytical framework positioned physical activity engagement as the primary exposure of interest, while systematically accounting for potential confounders through multivariable adjustment and sensitivity analyses.

**Fig. 2:**
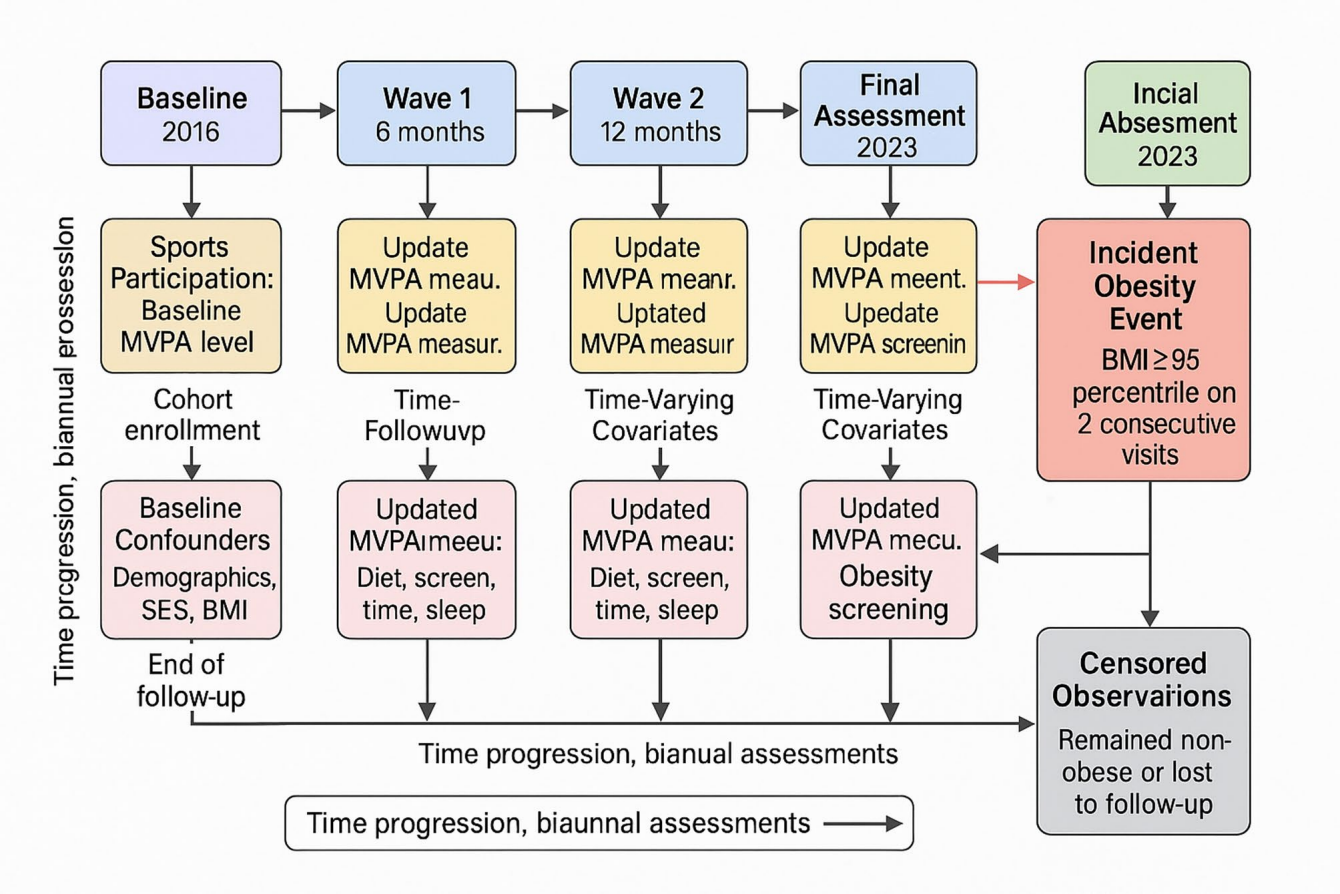
Conceptual framework of the cohort study design.

Figure 2 demonstrates the temporal architecture linking exposure assessment, covariate measurement, and outcome ascertainment across the follow-up trajectory. Our exposure variable—sports participation intensity—was operationalized through a composite index integrating frequency, duration, and metabolic intensity dimensions captured via the validated Chinese Adolescent Physical Activity Questionnaire (CAPAQ)^24^. Participants were classified into four hierarchical exposure categories at each assessment wave: (1) high participation, defined as structured moderate-to-vigorous physical activity exceeding 300 min weekly; (2) moderate participation, encompassing 150–300 min weekly; (3) low participation, representing 60–150 min weekly; and (4) minimal participation, denoting less than 60 min of weekly structured exercise. This categorization aligns with pediatric physical activity guidelines and provides sufficient granularity to detect dose-response gradients^25^.

The outcome of interest—incident obesity—was ascertained through age- and sex-specific BMI percentiles calculated at each follow-up wave using the WHO AnthroPlus reference standards. Obesity was defined as BMI-for-age exceeding the 95th percentile on two consecutive assessments, a requirement intended to enhance diagnostic specificity and minimize transient fluctuations^26^. Time-to-event analyses treated the first occasion meeting this criterion as the incident event, with participants remaining obesity-free classified as censored observations.

Covariate selection followed a directed acyclic graph approach to identify confounding pathways requiring statistical adjustment. Baseline covariates encompassed demographic factors (age, sex, urban/rural residence), socioeconomic indicators (parental education, household income quintile), anthropometric status (baseline BMI z-score), and family medical history (parental obesity, diabetes mellitus). Time-varying covariates measured at each wave included dietary quality assessed through the Youth Healthy Eating Index, daily screen time duration, sleep quantity, and pubertal development stage according to Tanner criteria^27^.

**Table 2:** Definitions and measurement methods for key study variables.

Variable	Definition	Measurement tool	Assessment frequency	Data source
Sports participation (exposure)	Weekly structured MVPA duration	CAPAQ questionnaire	Biannual	School survey
BMI-for-age percentile	Sex- and age-adjusted BMI rank	WHO AnthroPlus calculator	Biannual	Health examination
Incident obesity (outcome)	BMI ≥ 95th percentile on 2 + occasions	Physical measurement	Biannual	Health examination
Age	Years since birth	Administrative records	Baseline	School registry
Sex	Biological male/female	Administrative records	Baseline	School registry
Residence	Urban vs. rural classification	Administrative records	Baseline	School registry
Parental education	Highest degree attained	Self-report questionnaire	Baseline	Parent survey
Household income	Annual family income quintile	Self-report questionnaire	Baseline	Parent survey
Dietary quality	Youth Healthy Eating Index score	Food frequency questionnaire	Biannual	School survey
Screen time	Daily hours of recreational screen use	Self-report questionnaire	Biannual	School survey
Sleep duration	Average nightly sleep hours	Self-report questionnaire	Biannual	School survey
Pubertal stage	Tanner developmental classification	Self-assessment tool	Biannual	School survey

As presented in Table 2, we employed diverse assessment modalities including objective anthropometry, validated self-report instruments, and administrative data linkage. The measurement approaches balanced feasibility constraints inherent in large-scale cohort research against psychometric rigor, with most instruments demonstrating acceptable reliability coefficients (Cronbach’s α > 0.70) in prior validation studies.

### Statistical analysis methods

Descriptive statistics characterized baseline distributions and longitudinal patterns across exposure categories. Continuous variables were summarized as means with standard deviations or medians with interquartile ranges depending on distributional properties assessed via Shapiro–Wilk tests, while categorical variables were presented as frequencies and proportions. Between-group comparisons used analysis of variance for continuous measures and chi-square tests for categorical attributes.

The primary analytical approach was Cox proportional hazards regression, modeling time from baseline to incident obesity with sports participation level as the exposure of interest^28,29^. We selected this method because it naturally handles varying follow-up durations and censored observations—both common in longitudinal school-based studies where participants transfer or graduate at different times. The Cox model also accommodates time-varying covariates, which was essential given that dietary habits, screen time, and pubertal stage changed across adolescence in ways that simultaneously influenced activity patterns and obesity risk. The hazard function at time t for individual i can be expressed as h_i_(t) = h₀(t) × exp(β₁X₁_i_ + β₂X₂_i_ + ··· + βₚXₚ_i_), where h₀(t) is the baseline hazard and X₁ through Xₚ denote covariates. The proportional hazards assumption was evaluated through Schoenfeld residual tests and graphical assessment of log–log survival plots. We constructed sequential models: Model 1 adjusted for demographic factors only; Model 2 added socioeconomic covariates; Model 3 incorporated time-varying behavioral variables.

To address potential selection bias arising from systematic differences between highly active and minimally active adolescents, we implemented propensity score matching (PSM)^30^. We chose PSM because conventional multivariable regression relies on correct model specification across the entire covariate space, which becomes increasingly difficult with numerous confounders. PSM instead reduces the dimensionality problem by summarizing all measured confounders into a single scalar, and matching on this score creates comparison groups that resemble those produced by random assignment—thereby strengthening the basis for causal inference. The propensity for high physical activity engagement was estimated through logistic regression: logit(e_i_) = α₀ + α₁Z₁_i_ + ··· + αₖZₖ_i_, where e_i_ denotes the probability of high sports participation and Z₁ through Zₖ represent baseline confounders. Nearest-neighbor matching with a caliper width of 0.02 standard deviations of the logit propensity score was applied, and covariate balance post-matching was verified through standardized mean differences (values below 0.10 indicating adequate balance).

Stratified analyses examined whether associations varied across subgroups defined by sex, baseline BMI category, residential location, and socioeconomic status. Effect modification was tested through product interaction terms in Cox models. Sensitivity analyses probed robustness through varied obesity definitions, altered exposure categorization schemes, restriction to complete-case data, and competing risk models treating school dropout as a competing event. We also calculated E-values to quantify the minimum strength of unmeasured confounding required to nullify observed associations^31^. The E-value for an observed HR is computed as E-value = HR + √(HR × (HR − 1)). All analyses were performed using R version 4.2.1 (R Foundation for Statistical Computing) with packages survival, MatchIt, and cmprsk. Statistical significance was set at a two-tailed α of 0.05, though we placed greater emphasis on effect size magnitudes and confidence interval precision than on dichotomous significance testing.

## Empirical results and analysis

### Descriptive statistical analysis

The final analytical cohort comprised 89,532 adolescents contributing 523,847 person-years of observation, with a median follow-up of 6.2 years (interquartile range: 4.8–7.0 years). Baseline age ranged from 12.0 to 15.9 years, centering around 13.4 years—a developmental window marked by rapid physiological transitions and crystallizing behavioral patterns. Geographic diversity spanned all major administrative regions, although eastern coastal provinces contributed disproportionately, reflecting population density gradients.

**Fig. 3:**
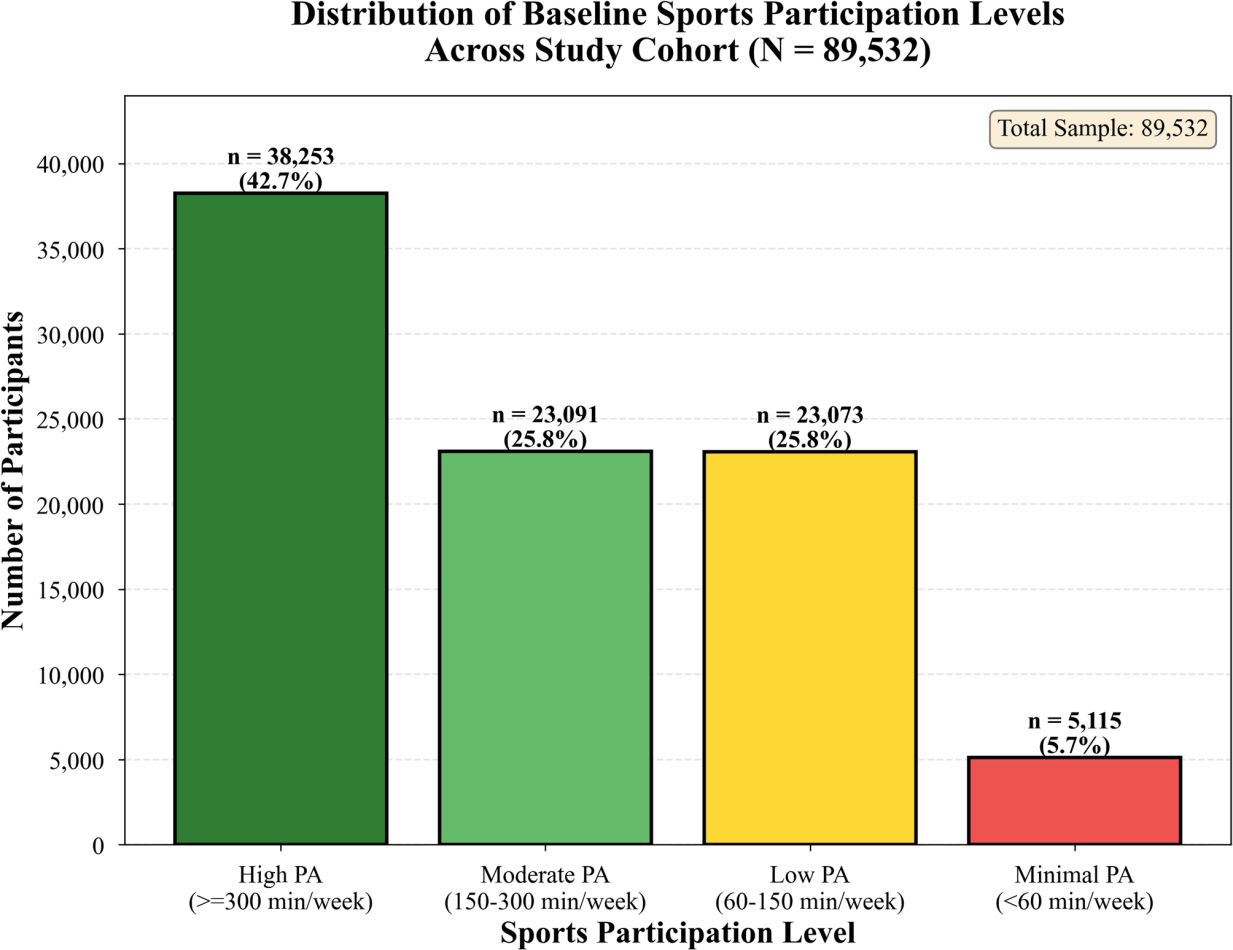
Distribution of baseline sports participation levels across the study cohort.

Figure 3 illustrates the baseline sports participation profile. High participation characterized 38,253 adolescents (42.7%) who reported structured moderate-to-vigorous activity exceeding 300 min weekly—a level consistent with optimal health recommendations^32^. Moderate participation encompassed 23,091 individuals (25.8%), low participation defined 23,073 (25.8%), and 5,115 adolescents (5.7%) fell into the minimal participation category. Distributions exhibited marked variation by sex, with boys demonstrating substantially higher participation rates at every intensity level.

**Table 3:** Baseline characteristics comparison across sports participation groups.

Characteristic	High PA (*n* = 38,253)	Moderate PA (*n* = 23,091)	Low PA (*n* = 23,073)	Minimal PA (*n* = 5,115)	*P*-value
Age, years (mean ± SD)	13.5 ± 1.1	13.4 ± 1.2	13.3 ± 1.1	13.2 ± 1.0	< 0.001
Male sex (%)	55.6	48.2	42.7	38.3	< 0.001
Urban residence (%)	72.4	68.9	64.2	58.7	< 0.001
Parental education ≥ college (%)	61.3	55.2	48.9	41.6	< 0.001
Household income > median (%)	56.8	51.4	47.3	43.2	< 0.001
BMI z-score (mean ± SD)	0.18 ± 0.82	0.31 ± 0.85	0.43 ± 0.89	0.52 ± 0.91	< 0.001
Dietary quality score (mean ± SD)	7.3 ± 1.8	6.9 ± 1.9	6.5 ± 2.0	6.1 ± 2.1	< 0.001
Screen time > 2 h/day (%)	39.7	46.8	52.9	61.4	< 0.001
Sleep duration, hours (mean ± SD)	8.4 ± 0.9	8.2 ± 0.9	8.1 ± 1.0	7.9 ± 1.0	< 0.001
Family history of obesity (%)	21.4	23.6	25.8	28.3	< 0.001

As presented in Table 3, high participation individuals tended to be slightly older, predominantly male, and more likely to reside in urban settings with greater access to recreational facilities^4^. Socioeconomic gradients emerged clearly, with parental educational attainment and household income positively associated with activity levels. Baseline BMI exhibited an inverse relationship with participation intensity—minimal activity participants carried mean BMI z-scores 0.34 units higher than their high-activity counterparts, though all groups remained within normal weight ranges by design. Behavioral covariates revealed clustering: active adolescents demonstrated superior dietary quality, reduced screen time, and more adequate sleep duration^33^.

During the observation period, 8,947 incident obesity cases occurred, yielding an overall incidence rate of 17.1 per 1,000 person-years. Annual rates escalated progressively during the initial three follow-up years before plateauing, possibly reflecting pubertal timing effects and adiposity rebound. Sex-specific incidence diverged markedly: boys exhibited 14.8 per 1,000 person-years compared with 19.3 per 1,000 person-years among girls—a reversal of typical childhood patterns likely reflecting differential pubertal fat redistribution^34^.

**Fig. 4:**
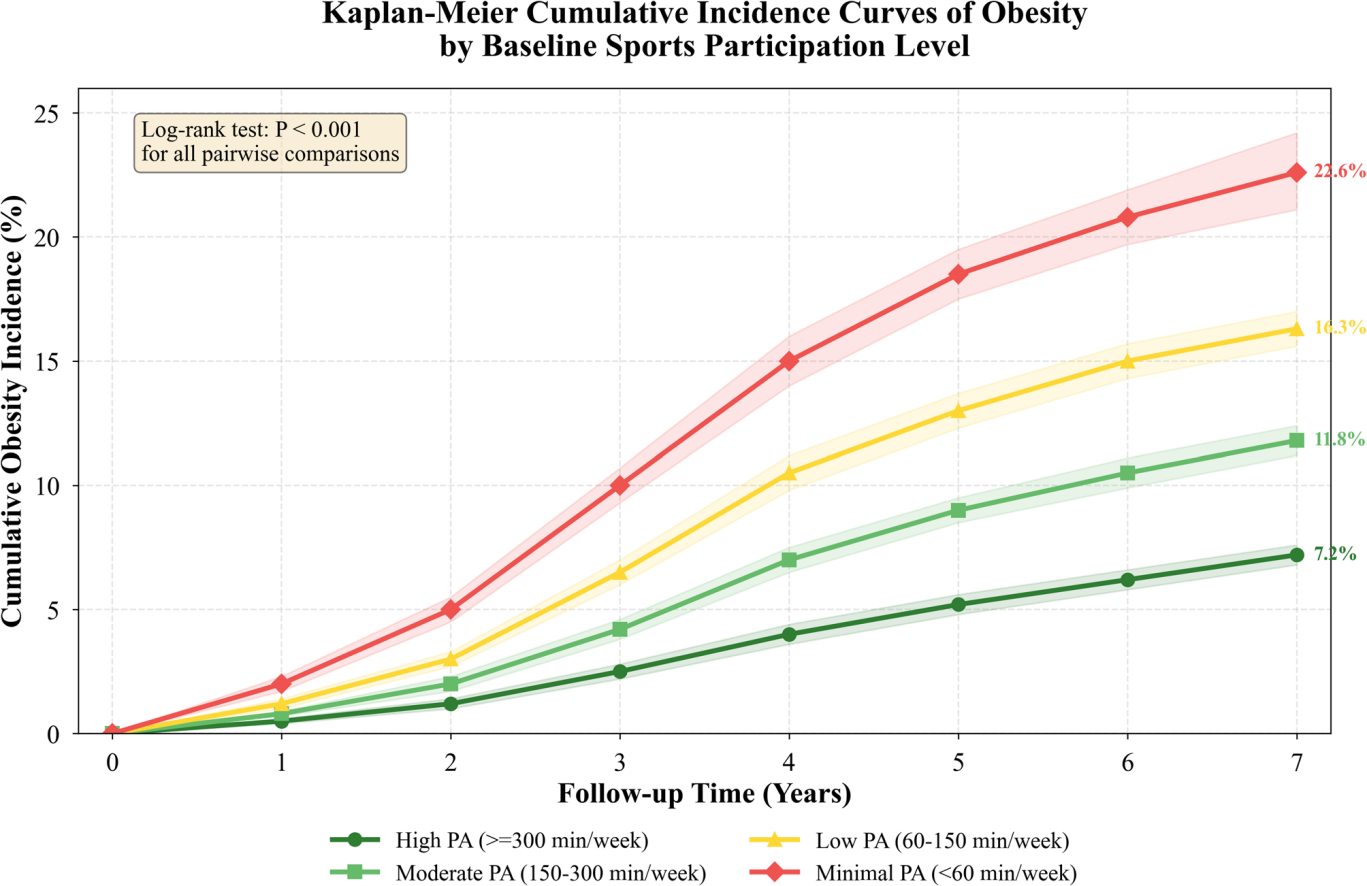
Kaplan–Meier cumulative incidence curves of obesity by baseline sports participation level.

Figure 4 displays cumulative obesity incidence trajectories. By seven years, cumulative incidence reached 7.2% (95% CI 6.8–7.6%) in the high participation group, 11.8% (95% CI 11.2–12.4%) among moderate participants, 16.3% (95% CI 15.6–17.0%) in the low participation stratum, and 22.6% (95% CI 21.1–24.2%) for minimal activity individuals. Log-rank tests confirmed significant differences across all pairwise comparisons (*P* < 0.001). The curves separated most steeply during years 2–4, the period of peak pubertal development.

Missing data were modest, with 15.7% of participants lacking complete follow-up across all six assessment waves, primarily because of school transfers and residential relocation rather than health-related attrition. Characteristics of participants with incomplete follow-up were similar to those with complete data, suggesting a missing-at-random mechanism. We addressed missingness through multiple imputation with chained equations, generating 20 imputed datasets and pooling estimates according to Rubin’s rules^35^. Sensitivity analyses comparing complete-case and imputed results yielded substantively identical conclusions.

### Impact analysis of sports participation on obesity incidence

Univariate analyses established preliminary associations, with unadjusted hazard ratios showing monotonic gradients across exposure categories. Relative to minimal participation, high participation yielded HR = 0.52 (95% CI 0.47–0.58), moderate participation HR = 0.68 (95% CI 0.62–0.75), and low participation HR = 0.79 (95% CI 0.72–0.87). These crude estimates, however, did not account for confounding structures that might partially explain the observed associations.

**Table 4:** Cox regression analysis of sports participation effects on obesity incidence.

Exposure category	Events/total	Incidence rate	Model 1 h (95% CI)	Model 2 h (95% CI)	Model 3 h (95% CI)	*P*-value
Minimal PA	1156/5,115	31.4 per 1000 PY	1.00 (Reference)	1.00 (Reference)	1.00 (Reference)	–
Low PA	3761/23,073	21.7 per 1000 PY	0.76 (0.69–0.83)	0.74 (0.68–0.81)	0.73 (0.66–0.81)	< 0.001
Moderate PA	2277/23,091	13.2 per 1000 PY	0.64 (0.58–0.71)	0.62 (0.56–0.69)	0.61 (0.54–0.68)	< 0.001
High PA	1753/38,253	6.1 per 1000 PY	0.48 (0.43–0.54)	0.44 (0.39–0.50)	0.42 (0.37–0.48)	< 0.001
Per 100 min/week increase	–	–	0.88 (0.86–0.90)	0.87 (0.85–0.89)	0.86 (0.84–0.88)	< 0.001
Frequency (per session/week)	–	–	0.91 (0.89–0.93)	0.90 (0.88–0.92)	0.89 (0.87–0.91)	< 0.001
Intensity (moderate vs. light)	–	–	0.72 (0.68–0.76)	0.71 (0.67–0.75)	0.69 (0.65–0.73)	< 0.001
Intensity (vigorous vs. light)	–	–	0.58 (0.54–0.62)	0.56 (0.52–0.60)	0.54 (0.50–0.58)	< 0.001

PY, person-years; PA, physical activity; HR, hazard ratio; CI, confidence interval. Model 1: adjusted for age, sex, and residence. Model 2: Model 1 + parental education and household income. Model 3: Model 2 + time-varying dietary quality, screen time, sleep duration, and pubertal stage.

As presented in Table 4, the fully-adjusted Model 3 showed that high sports participation reduced the obesity hazard by 58% relative to minimal activity (HR = 0.42, 95% CI 0.37–0.48, *P* < 0.001). Moderate and low participation demonstrated intermediate protection (HR = 0.61 and 0.73, respectively), establishing a clear dose-response gradient^36^. The proportional hazards assumption held reasonably well, though modest violations were detected for baseline BMI z-score (Schoenfeld test *P* = 0.031), which we addressed by stratifying on baseline BMI category.

**Fig. 5:**
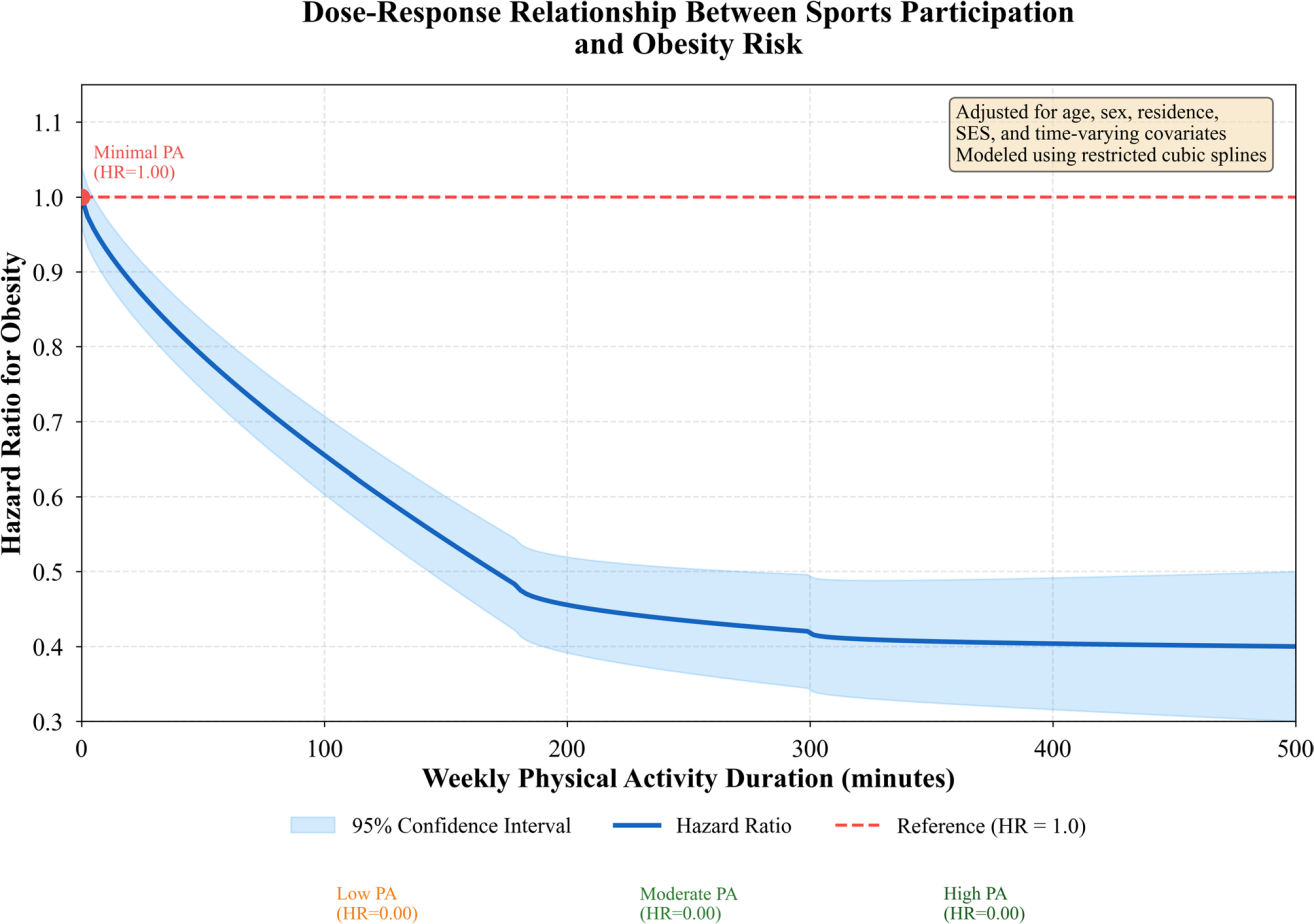
Dose-response relationship between sports participation and obesity risk.

Figure 5 presents the dose-response curve derived through restricted cubic splines with knots at the 10th, 50th, and 90th percentiles^37^. The association was nonlinear: steep risk reductions were apparent from 0 to approximately 200 min weekly, beyond which the gradient moderated. The inflection point occurred near 180 min per week, corresponding to consensus guidelines recommending 150–180 min of moderate-to-vigorous activity for adolescents. Incremental benefits persisted at higher volumes, though with diminishing marginal returns consistent with physiological principles of energy balance and metabolic adaptation^38^.

Activity frequency demonstrated significant protective effects independent of total duration, with each additional session per week associated with 11% lower obesity risk (HR = 0.89, 95% CI 0.87–0.91). Exercise intensity proved particularly consequential: vigorous-intensity activities yielded a 46% risk reduction compared with light-intensity pursuits at equivalent durations (HR = 0.54, 95% CI 0.50–0.58), in line with evidence that high-intensity exercise promotes greater post-exercise oxygen consumption^39^.

Time-varying confounders evolved across adolescence in ways that both influenced subsequent activity levels and predicted obesity risk, creating complex feedback structures. Marginal structural models with inverse probability weighting were used to account for these time-dependent relationships. Stabilized weights had a mean of 1.03 (range: 0.28–3.81), indicating reasonable positivity. Results from weighted models yielded HR = 0.44 (95% CI 0.38–0.51) for high versus minimal participation, paralleling the standard adjustment results.

**Fig. 6:**
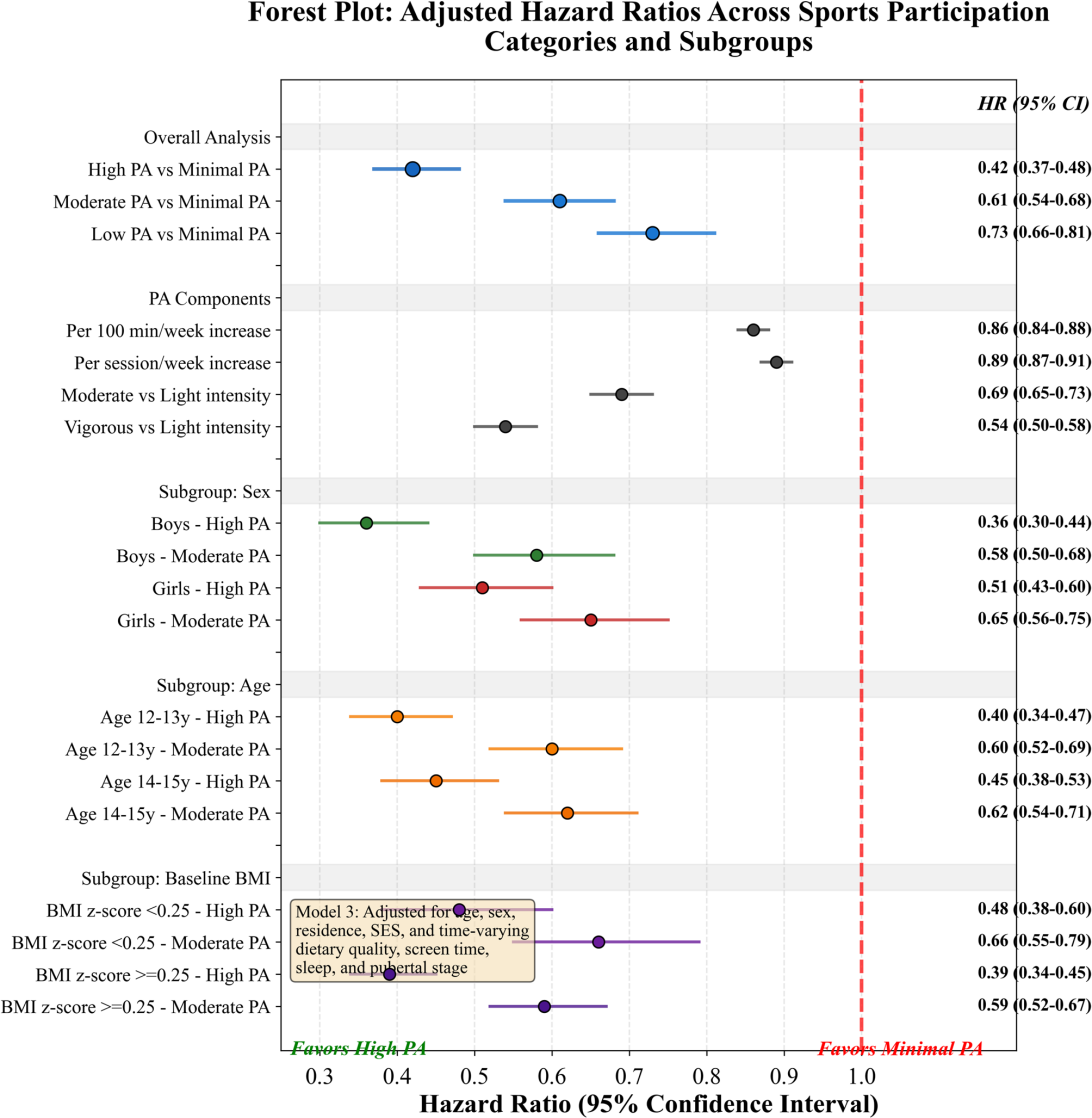
Forest plot of adjusted hazard ratios across sports participation categories and subgroups.

Figure 6 displays the fully-adjusted hazard ratios alongside subgroup analyses. In absolute terms, high sports participation reduced seven-year obesity incidence from 22.6% to 7.2%, yielding a number needed to treat of 6.5 adolescents engaging in high-level activity for seven years to prevent one obesity case^40^. E-value calculations indicated that an unmeasured confounder would need to produce a hazard ratio of 3.47 with both exposure and outcome to fully explain the observed association—a threshold exceeding most plausible confounding scenarios^41^.

### Heterogeneity and robustness testing

Subgroup analyses examined whether protective associations varied across population strata (Table 5). Sex emergedas a signifi cant eff ect modifi er (P-interaction = 0.007): among boys, high versus minimal participation yielded HR =0.36 (95% CI 0.30–0.44), whereas girls showed HR = 0.51 (95% CI 0.43–0.60) ^42^. This sex diff erential may refl ecthormonal infl uences on metabolism and body composition, or social determinants shaping activity preferencesbetween sexes. Age at baseline did not modify associations substantially (P-interaction = 0.284). Stratifi cation bybaseline BMI z-score ( 0.25 vs ≥ 0.25) revealed a signifi cant interaction (P-interaction = 0.042), with adolescents inthe higher BMI z-score stratum deriving a stronger relative protective eff ect (HR = 0.39, 95% CI 0.34–0.45) thanthose in the lower stratum (HR = 0.48, 95% CI 0.38–0.60).

**Table 5:** Subgroup analyses and robustness testing results summary.

Subgroup/analysis	*n*	Events	High PA HR (95% CI)	Moderate PA HR (95% CI)	*P*-interaction	E-value	Interpretation
Boys	43,586	4127	0.36 (0.30–0.44)	0.58 (0.50–0.68)	0.007	4.12	Stronger effect in boys
Girls	45,946	4820	0.51 (0.43–0.60)	0.65 (0.56–0.75)	–	2.85	Moderate effect in girls
Age 12–13 years	48,326	4516	0.40 (0.34–0.47)	0.60 (0.52–0.69)	0.284	3.58	No age modification
Age 14–15 years	41,206	4431	0.45 (0.38–0.53)	0.62 (0.54–0.71)	–	3.24	Consistent across ages
BMI z-score < 0.25	29,845	2189	0.48 (0.38–0.60)	0.66 (0.55–0.79)	0.042	3.01	Weaker relative effect
BMI z-score ≥ 0.25	59,687	6758	0.39 (0.34–0.45)	0.59 (0.52–0.67)	–	3.71	Stronger relative effect
PSM-matched cohort	20,460	2834	0.44 (0.37–0.52)	0.63 (0.54–0.73)	–	3.32	Consistent after PSM
Alt. obesity definition	89,532	11234	0.39 (0.35–0.44)	0.58 (0.53–0.64)	–	3.71	Robust to definition
Excl. first-year events	87,913	7265	0.41 (0.36–0.48)	0.60 (0.53–0.68)	–	3.53	No reverse causation

PA, physical activity; HR, hazard ratio; CI, confidence interval; PSM, propensity score matching; BMI, body mass index.

**Fig. 7:**
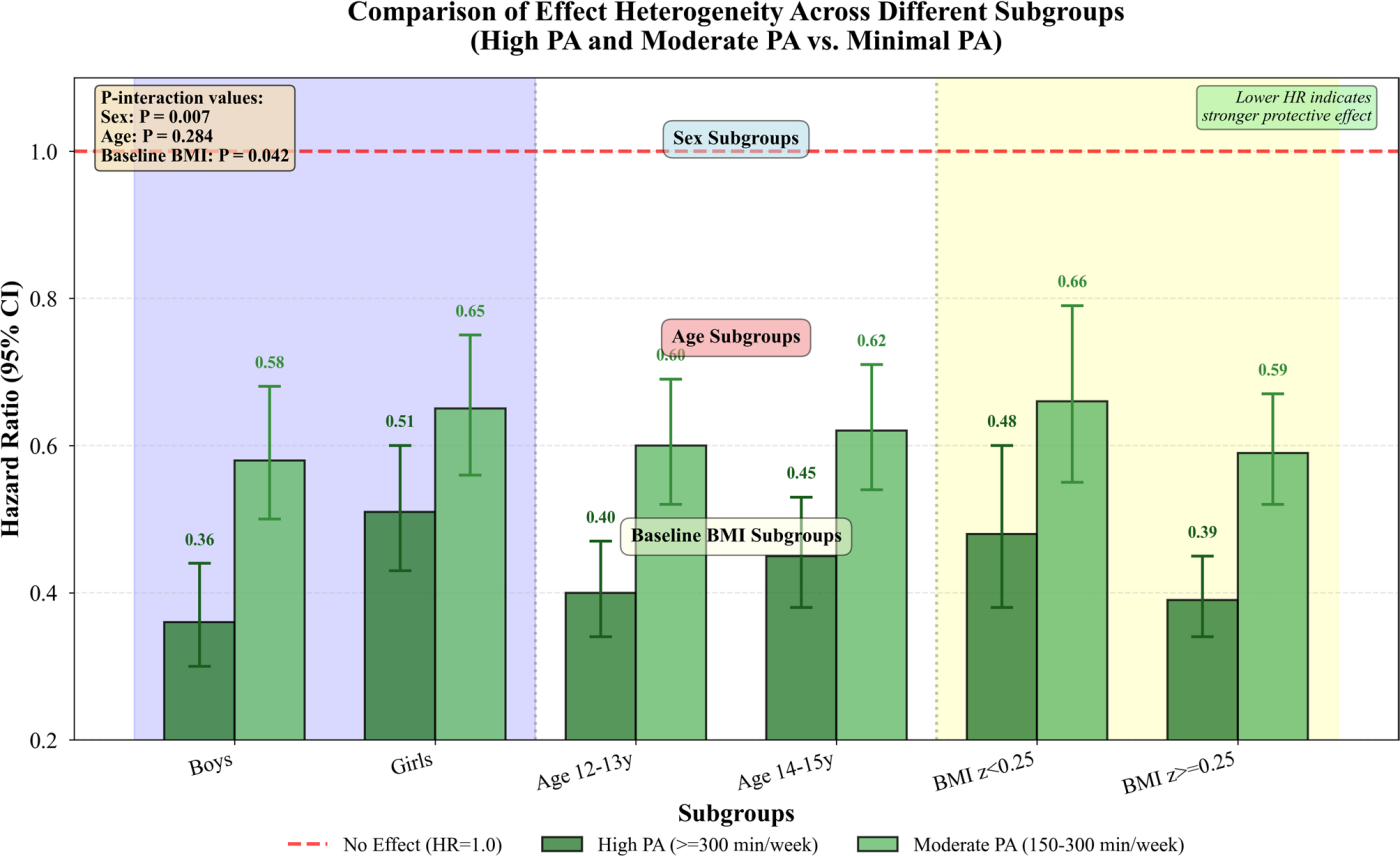
Comparison of effect heterogeneity across different subgroups.

Figure 7 illustrates hazard ratios for high sports participation across pre-specified subgroups. While point estimates varied, confidence intervals overlapped substantially across most strata, suggesting that the core protective association generalizes broadly. The notable exception was sex-specific effects, where confidence intervals for boys and girls showed minimal overlap, supporting genuine effect modification.

Propensity score matching created a balanced cohort of 20,460 participants (10,230 per exposure group) with all standardized mean differences below 0.05^43^. Cox regression within this matched cohort yielded HR = 0.44 (95% CI 0.37–0.52), nearly identical to unmatched adjusted estimates, bolstering causal inference validity. Robustness testing with alternative obesity definitions (single-occasion and combined waist circumference criteria) identified 11,234 cases, with hazard ratios for high participation ranging from 0.39 to 0.46. Lag analyses excluding first-year events yielded HR = 0.41 (95% CI 0.36–0.48), arguing against substantial reverse causation^44^.

**Fig. 8:**
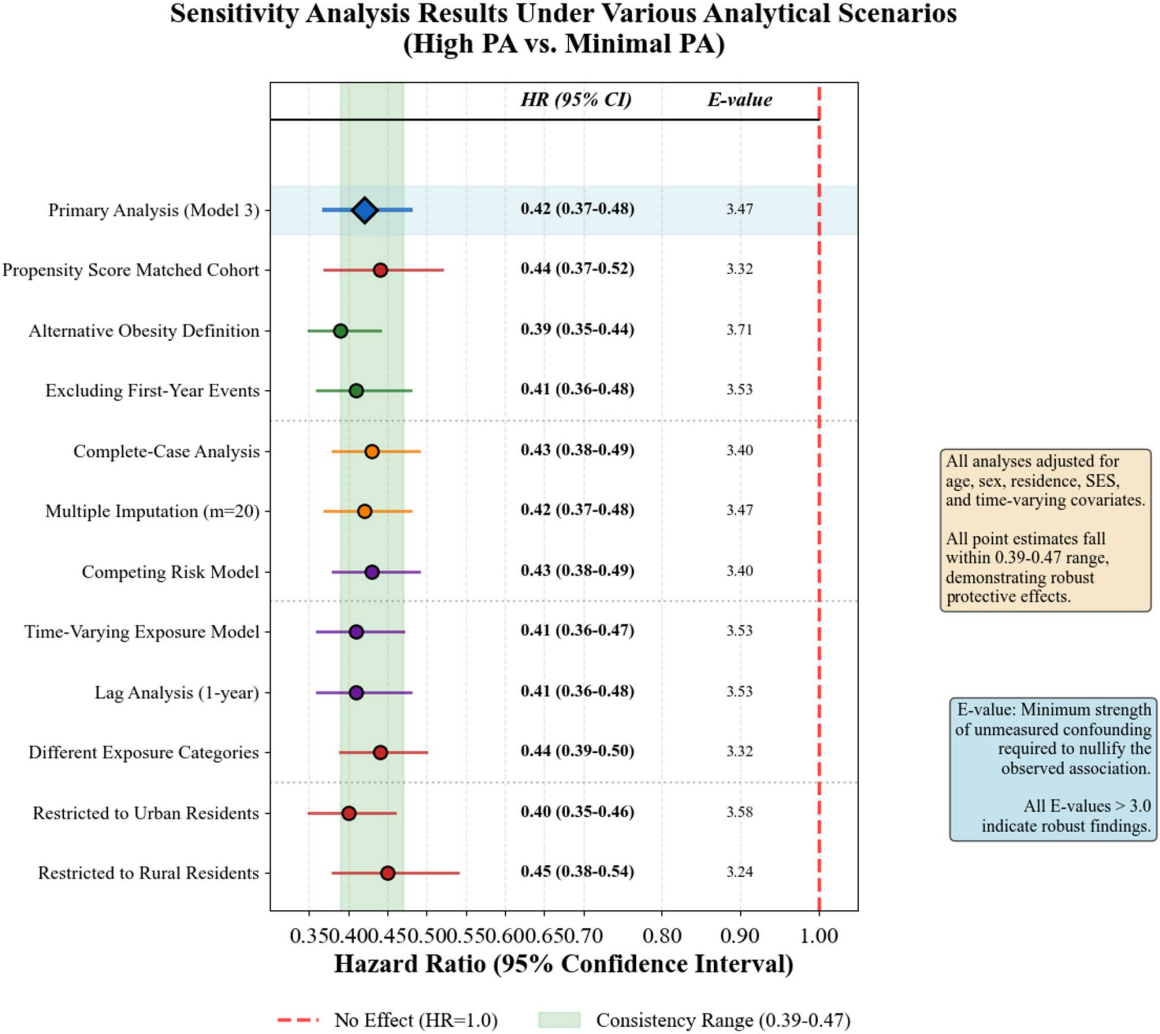
Sensitivity analysis results under various analytical scenarios.

Figure 8 displays hazard ratios under multiple sensitivity scenarios. Point estimates ranged between 0.39 and 0.47, and E-values exceeded 3.0 across all analyses, indicating that any unmeasured confounder would require implausibly strong associations with both exposure and outcome to nullify the observed effects.

## Discussion

This large-scale prospective cohort investigation established that sustained sports participation during adolescence substantially reduces obesity incidence, with protective effects demonstrating clear dose-response gradients and persisting after comprehensive confounder adjustment. High-intensity physical activity engagement lowered obesity risk by 58% compared with minimal participation across seven years of follow-up, translating to absolute risk reductions of 15.4% points. These findings remained consistent across multiple sensitivity analyses and analytical approaches, strengthening causal inference despite the observational design.

Our results broadly align with prior epidemiological evidence while extending understanding in several directions. A meta-analysis of 37 cohort studies reported pooled relative risks of 0.66 for high versus low physical activity in relation to childhood obesity—somewhat more conservative than our HR of 0.42^45^. This discrepancy likely stems from our use of objective anthropometric measurements, longer follow-up, and more rigorous statistical adjustment. The dose-response pattern we documented—steep risk reductions up to approximately 180 min weekly followed by attenuating marginal benefits—parallels findings from accelerometer-based studies in European adolescents, although earlier investigations rarely achieved sufficient sample sizes to model nonlinear relationships with precision^46^.

The biological plausibility of our findings rests on established mechanisms linking physical activity to energy homeostasis and metabolic regulation. Structured exercise increases total energy expenditure both acutely through muscle contraction and chronically via elevated resting metabolic rate resulting from increased lean body mass. Beyond thermodynamic effects, regular activity improves insulin sensitivity, modulates appetite-regulating hormones, and may influence epigenetic programming of adipocyte development—processes that appear particularly malleable during adolescence^47^. The stronger protective effects we observed for vigorous-intensity activities likely stem from greater excess post-exercise oxygen consumption and metabolic perturbation following high-intensity bouts.

Our analyses uncovered important heterogeneity across population subgroups. Boys derived greater obesity risk reductions than girls from equivalent activity volumes, potentially reflecting sex differences in body composition during puberty, sociocultural factors shaping activity preferences, or differential metabolic responses to exercise. The observed effect modification by baseline BMI suggests that adolescents already trending toward the upper boundary of normal weight may constitute an especially responsive target population for activity-based interventions. These findings underscore the need to tailor prevention strategies to specific demographic and risk profiles.

From a public health policy perspective, our results furnish quantitative support for efforts to expand adolescent physical activity opportunities through school-based programming, urban planning that facilitates active transportation, and community recreation infrastructure. The number needed to treat of 6.5 implies that even modest population-level increases in sports participation could yield substantial obesity prevention benefits when scaled across millions of adolescents. At the same time, effective policy must address the socioeconomic gradients in activity access we documented—cost, facility availability, and cultural norms disproportionately constrain disadvantaged populations^48^.

This study possesses several strengths: an exceptionally large sample providing statistical power for subgroup analyses, extended prospective follow-up ensuring temporal sequencing of exposure and outcome, standardized measurement protocols minimizing information bias, and sophisticated statistical methods addressing time-dependent confounding. Nevertheless, several limitations warrant acknowledgment. First, residual confounding from unmeasured factors such as genetic predisposition remains possible, despite high E-values suggesting robustness. Second, physical activity assessment relied partially on self-report instruments, which, though validated, introduce measurement error relative to objective accelerometry. Third, the generalizability of findings from Chinese adolescents to populations with different ethnic compositions, obesity prevalence patterns, and activity cultures requires empirical verification. Fourth, although our seven-year follow-up represents a substantial improvement over most existing cohorts, extended observation into young adulthood would reveal whether adolescent activity patterns establish durable obesity resistance or merely delay onset.

Future research should prioritize randomized controlled trials of structured activity interventions powered to detect obesity incidence effects, even though such designs face considerable logistical and ethical challenges. Investigations incorporating metabolomic and genomic profiling could elucidate individual-level response heterogeneity and identify biomarkers predicting intervention responsiveness^49^. The evolving landscape of digital health technologies offers promising avenues for delivering personalized activity promotion at scale while generating granular behavioral data that surpass traditional assessment methods.

## Conclusion

This investigation represents one of the most comprehensive examinations to date of the longitudinal relationship between adolescent sports participation patterns and obesity incidence, tracking nearly 90,000 Chinese adolescents across seven years. Through rigorous prospective cohort methodology and advanced statistical techniques—including Cox proportional hazards regression, propensity score matching, and marginal structural models—we established that sustained physical activity engagement confers substantial, dose-dependent protection against obesity development during this critical life stage.

Our principal findings show that high-intensity sports participation reduces obesity risk by 58% compared with minimal activity after accounting for demographic, socioeconomic, and behavioral confounders. This protective association persists across multiple sensitivity analyses and exhibits nonlinear dose-response characteristics, with the steepest risk reductions occurring between 0 and 180 min of weekly moderate-to-vigorous activity. Activity frequency and intensity independently contribute to obesity prevention, suggesting that distributing exercise across multiple weekly sessions and pursuing vigorous-intensity activities optimize metabolic benefits. Sex-specific analyses revealed stronger protective effects among boys, highlighting the need for tailored intervention approaches that consider biological and sociocultural heterogeneity.

Several limitations temper our conclusions. Residual confounding from unmeasured genetic or environmental factors cannot be entirely excluded. Reliance on validated questionnaires rather than objective accelerometry introduces some measurement error. The ethnic and geographic focus on Chinese adolescents constrains direct generalizability to other populations. Despite these caveats, the consistency of our findings across numerous analytical strategies and the high E-values bolster confidence in the robustness of the observed associations.

In conclusion, this study provides compelling evidence that promoting adolescent sports participation represents a viable, evidence-based strategy for curbing the obesity epidemic. The dose-response relationships we documented offer concrete guidance for physical activity guideline development, while the identified effect modifiers illuminate opportunities for targeted prevention among high-risk subgroups. We reported this study in accordance with the STROBE guidelines for cohort studies.

## Data Availability

The datasets generated and analyzed during the current study are available from the corresponding author (Chunmin Dai, dcm075213@163.com) upon reasonable request. Access to data is subject to data sharing agreements and institutional review board approval to ensure participant confidentiality and compliance with data protection regulations.

## Abbreviations

ATE: Average treatment effect
BMI: Body mass index
CAPAQ: Chinese adolescent physical activity questionnaire
CI: Confidence interval
HR: Hazard ratio
IPAQ: International physical activity questionnaire
METs: Metabolic equivalent of task
MSM: Marginal structural models
MVPA: Moderate-to-vigorous physical activity
NAHSS: National adolescent health surveillance system
PA: Physical activity
PAQ-A: Physical activity questionnaire for adolescents
PSM: Propensity score matching
PY: Person-years
SD: Standard deviation
STROBE: Strengthening the reporting of observational studies in epidemiology
WHO: World Health Organization

## References

[1] NCD Risk Factor Collaboration. Worldwide trends in underweight and obesity from 1990 to 2022: a pooled analysis of 3663 population-representative studies with 222 million children, adolescents, and adults. *Lancet* **403**(10431), 1027–1050 (2024). 38432237 10.1016/S0140-6736(23)02750-2 PMC7615769

[2] Zhang, X. et al. Global prevalence of overweight and obesity in children and adolescents: A systematic review and meta-analysis. *JAMA Pediatr.* **178**(7), 642–653 (2024). 10.1001/jamapediatrics.2024.1576 PMC11165417 38856986

[3] Yuan, Z. et al. Control of childhood obesity and implications for policy in China. *Lancet Public. Health*. **9**(12), e1020–e1030 (2024). 10.1016/S2468-2667(24)00263-9 39579776

[4] Li, H. et al. Epidemiology of obesity and influential factors in China: A multicenter cross-sectional study of children and adolescents. *BMC Pediatr.* **24**(1), 498 (2024). 39095721 10.1186/s12887-024-04970-1 PMC11295318

[5] Simmonds, M., Llewellyn, A., Owen, C. G. & Woolacott, N. Predicting adult obesity from childhood obesity: A systematic review and meta-analysis. *Obes. Rev.* **17**(2), 95–107 (2016). 26696565 10.1111/obr.12334

[6] Wyszyńska, J. et al. Physical activity in the prevention of childhood obesity: The position of the European Childhood Obesity Group and the European Academy of Pediatrics. *Front. Pediatr.* **8**, 535 (2020). 33224905 10.3389/fped.2020.535705 PMC7674497

[7] Jurado-Castro, J. M., Gil-Campos, M., Gonzalez-Gonzalez, H. & Llorente-Cantarero, F. J. Evaluation of physical activity and lifestyle interventions focused on school children with obesity using accelerometry: A systematic review and meta-analysis. *Int. J. Environ. Res. Public. Health*. **17**(17), 6031 (2020). 32825085 10.3390/ijerph17176031 PMC7503305

[8] Lee, J. H. & Cho, J. Sleep and obesity. *Sleep. Med. Clin.* **17**(1), 111–116 (2022). 35216758 10.1016/j.jsmc.2021.10.009

[9] Shiba, K. & Kawahara, T. Using propensity scores for causal inference: Pitfalls and tips. *J. Epidemiol.* **31**(8), 457–463 (2021). 34121051 10.2188/jea.JE20210145 PMC8275441

[10] Li, T. & Li, C. Changes in weight distribution and trends in obesity among children and adolescents in East Asia: Insights from NCD-RisC data. *Anthropol. Anz*. **81**(2), 161–171 (2024). 39531456 10.1371/journal.pone.0310646 PMC11556726

[11] Ainsworth, B. E. et al. 2011 Compendium of Physical Activities: A second update of codes and MET values. *Med. Sci. Sports Exerc.* **43**(8), 1575–1581 (2011). 21681120 10.1249/MSS.0b013e31821ece12

[12] Tremblay, M. S. et al. Global matrix 2.0: Report card grades on the physical activity of children and youth comparing 38 countries. *J. Phys. Act. Health*. **13**(11 Suppl 2), S343–S366 (2016). 27848745 10.1123/jpah.2016-0594

[13] Hagströmer, M., Oja, P. & Sjöström, M. The International Physical Activity Questionnaire (IPAQ): A study of concurrent and construct validity. *Public. Health Nutr.* **9**(6), 755–762 (2006). 16925881 10.1079/phn2005898

[14] Sallis, J. F., Owen, N. & Fisher, E. Ecological models of health behavior. In (eds Glanz, K., Rimer, B. K. & Viswanath, K.) Health Behavior: Theory, Research, and Practice. 5th edn 43–64 (San Francisco, CA: Jossey-Bass, 2015).

[15] Calcaterra, V. et al. The use of the body mass index (BMI) in children and adolescents: Advantages and pitfalls. *Nutrients* **15**(12), 2764 (2023).37375668

[16] Bleich, S. N. et al. Interventions to prevent global childhood overweight and obesity: A systematic review. *Lancet Diabetes Endocrinol.* **6**(4), 332–346 (2018). 29066096 10.1016/S2213-8587(17)30358-3

[17] Afshin, A. et al. Health effects of overweight and obesity in 195 countries over 25 years. *N Engl. J. Med.* **377**(1), 13–27 (2017). 28604169 10.1056/NEJMoa1614362 PMC5477817

[18] Loux, T., Varghese, M., Romero, D. & Katta, B. Trends in U.S. adolescent physical activity and obesity: A 20-year age-period-cohort analysis. *Pediatr. Obes.* **18**(3), e12996 (2023). 36517961 10.1111/ijpo.12996

[19] Hernán, M. A. & Robins, J. M. Using big data to emulate a target trial when a randomized trial is not available. *Am. J. Epidemiol.* **183**(8), 758–764 (2016). 26994063 10.1093/aje/kwv254 PMC4832051

[20] Raghupathi, W. & Raghupathi, V. Big data analytics in healthcare: Promise and potential. *Health Inf. Sci. Syst.* **2**, 3 (2014). 25825667 10.1186/2047-2501-2-3 PMC4341817

[21] Hong, Y., Ullah, R., Wang, J. B. & Fu, J. F. Trends of obesity and overweight among children and adolescents in China. *World J. Pediatr.* **19**(12), 1115–1126 (2023). 36920656 10.1007/s12519-023-00709-7 PMC10015139

[22] de Onis, M. et al. Development of a WHO growth reference for school-aged children and adolescents. *Bull. World Health Organ.* **85**(9), 660–667 (2007). 18026621 10.2471/BLT.07.043497 PMC2636412

[23] Lohman, T. G., Roche, A. F. & Martorell, R. *Anthropometric Standardization Reference Manual*(Human Kinetics Books, 1988).

[24] Wang, C., Chen, P. & Zhuang, J. Validity and reliability of International Physical Activity Questionnaire-Short Form in Chinese youth. *Res. Q. Exerc. Sport*. **84**(Suppl 2), S80–S86 (2013). 24527570 10.1080/02701367.2013.850991

[25] Bull, F. C. et al. World Health Organization 2020 guidelines on physical activity and sedentary behaviour. *Br. J. Sports Med.* **54**(24), 1451–1462 (2020). 33239350 10.1136/bjsports-2020-102955 PMC7719906

[26] Cole, T. J. & Lobstein, T. Extended international (IOTF) body mass index cut-offs for thinness, overweight and obesity. *Pediatr. Obes.* **7**(4), 284–294 (2012). 22715120 10.1111/j.2047-6310.2012.00064.x

[27] Hirshkowitz, M. et al. National Sleep Foundation’s sleep time duration recommendations: Methodology and results summary. *Sleep. Health*. **1**(1), 40–43 (2015). 29073412 10.1016/j.sleh.2014.12.010

[28] Tarkhan, A. & Simon, N. An online framework for survival analysis: reframing Cox proportional hazards model for large data sets and neural networks. *Biostatistics* **25**(1), 134–153 (2024). 10.1093/biostatistics/kxac039 PMC10724274 36288541

[29] Cox, D. R. Regression models and life-tables. *J. R Stat. Soc. Ser. B Stat. Methodol.* **34**(2), 187–220 (1972).

[30] Lanza, S. T., Moore, J. E. & Butera, N. M. Drawing causal inferences using propensity scores: A practical guide for community psychologists. *Am. J. Community Psychol.* **52**(3–4), 380–392 (2013). 24185755 10.1007/s10464-013-9604-4 PMC4098642

[31] VanderWeele, T. J. & Ding, P. Sensitivity analysis in observational research: Introducing the E-value. *Ann. Intern. Med.* **167**(4), 268–274 (2017). 28693043 10.7326/M16-2607

[32] Migueles, J. H. et al. Accelerometer data collection and processing criteria to assess physical activity and other outcomes: A systematic review and practical considerations. *Sports Med.* **47**(9), 1821–1845 (2017). 28303543 10.1007/s40279-017-0716-0 PMC6231536

[33] Chaput, J. P. et al. 2020 WHO guidelines on physical activity and sedentary behaviour for children and adolescents aged 5–17 years: Summary of the evidence. *Int. J. Behav. Nutr. Phys. Act.* **17**(1), 141 (2020). 33239009 10.1186/s12966-020-01037-z PMC7691077

[34] Fryar, C. D., Carroll, M. D. & Afful, J. *Prevalence of overweight, obesity, and severe obesity among children and adolescents aged 2–19 years: United States, 1963–1965 through 2017–2018* (NCHS Health E-Stats, 2020).

[35] Rubin, D. B. *Multiple Imputation for Nonresponse in Surveys* (Wiley, 1987).

[36] Pan, X. F., Wang, L. & Pan, A. Epidemiology and determinants of obesity in China. *Lancet Diabetes Endocrinol.* **9**(6), 373–392 (2021). 34022156 10.1016/S2213-8587(21)00045-0

[37] Desquilbet, L. & Mariotti, F. Dose-response analyses using restricted cubic spline functions in public health research. *Stat. Med.* **29**(9), 1037–1057 (2010). 20087875 10.1002/sim.3841

[38] LaForgia, J., Withers, R. T. & Gore, C. J. Effects of exercise intensity and duration on the excess post-exercise oxygen consumption. *J. Sports Sci.* **24**(12), 1247–1264 (2006). 17101527 10.1080/02640410600552064

[39] Egan, B. & Zierath, J. R. Exercise metabolism and the molecular regulation of skeletal muscle adaptation. *Cell. Metab.* **17**(2), 162–184 (2013). 23395166 10.1016/j.cmet.2012.12.012

[40] Rose, G. Sick individuals and sick populations. *Int. J. Epidemiol.* **14**(1), 32–38 (1985). 3872850 10.1093/ije/14.1.32

[41] Mathur, M. B., Ding, P., Riddell, C. A. & VanderWeele, T. J. Website and R package for computing E-values. *Epidemiology* **29**(5), e45–e47 (2018). 29912013 10.1097/EDE.0000000000000864 PMC6066405

[42] Yang, X., Telama, R., Viikari, J. & Raitakari, O. T. Risk of obesity in relation to physical activity tracking from youth to adulthood. *Med. Sci. Sports Exerc.* **38**(5), 919–925 (2006). 16672846 10.1249/01.mss.0000218121.19703.f7

[43] Austin, P. C. An introduction to propensity score methods for reducing the effects of confounding in observational studies. *Multivar. Behav. Res.* **46**(3), 399–424 (2011). 10.1080/00273171.2011.568786 PMC3144483 21818162

[44] Patino, C. M. & Ferreira, J. C. Sensitivity analysis: Beyond the always optimistic best case/worst case scenario. *J. Bras. Pneumol*. **44**(4), 259 (2018).30328923

[45] Poitras, V. J. et al. Systematic review of the relationships between objectively measured physical activity and health indicators in school-aged children and youth. *Appl. Physiol. Nutr. Metab.* **41**(6 Suppl 3), S197–S239 (2016). 27306431 10.1139/apnm-2015-0663

[46] Liu, Z. et al. A systematic review and meta-analysis of the overall effects of school-based obesity prevention interventions and effect differences by intervention components. *Int. J. Behav. Nutr. Phys. Act.* **16**(1), 95 (2019). 31665040 10.1186/s12966-019-0848-8 PMC6819386

[47] Xu, S. & Xue, Y. Pediatric obesity: Causes, symptoms, prevention and treatment. *Exp. Ther. Med.* **11**(1), 15–20 (2016). 26834850 10.3892/etm.2015.2853 PMC4726862

[48] Backholer, K. et al. Differential exposure to, and potential impact of, unhealthy advertising to children by socio-economic and ethnic groups: A systematic review of the evidence. *Obes. Rev.* **22**(3), e13144 (2021). 33073488 10.1111/obr.13144

[49] Siddiqui, S. T., Kandala, N. B. & Stranges, S. Use of machine learning algorithms for precision oncology, genomics, metabolomics and pharmacogenomics: A systematic review. *BMC Med. Inf. Decis. Mak.* **22**(1), 235 (2022).

